# The Cytoplasmic Tail of Ovine Herpesvirus 2 Glycoprotein B Affects Cell Surface Expression and Is Required for Membrane Fusion

**DOI:** 10.3390/v17070994

**Published:** 2025-07-16

**Authors:** Colleen M. Lynch, Maria K. Herndon, McKenna A. Hull, Daniela D. Moré, Katherine N. Baker, Cristina W. Cunha, Anthony V. Nicola

**Affiliations:** 1 Department of Veterinary Microbiology and Pathology, College of Veterinary Medicine, Washington State University, Pullman, WA 99164, USA; colleen.lynch@wsu.edu (C.M.L.); mkmeyer@wsu.edu (M.K.H.); mckenna.hull@wsu.edu (M.A.H.); daniela.d.more@wsu.edu (D.D.M.); 2 Animal Disease Research Unit, Agricultural Research Service, United States Department of Agriculture, Pullman, WA 99164, USA; katherine.baker@usda.gov

**Keywords:** ovine herpesvirus 2, glycoprotein B, surface expression, fusion

## Abstract

Ovine herpesvirus 2 (OvHV-2) causes the fatal veterinary disease malignant catarrhal fever (MCF). Fusion is an essential step in the host cell entry of enveloped viruses and is an important target for vaccine development. OvHV-2 cannot be propagated in vitro, so a robust virus-free cell–cell membrane fusion assay is necessary to elucidate its entry mechanism. OvHV-2 cell–cell fusion requires three conserved herpesviral envelope glycoproteins: gB, gH, and gL. OvHV-2 fusion activity is detectable but low. We hypothesize that enhancing the cell surface expression of gB, which is the core herpesviral fusogen, will increase cell–cell fusion. We generated C-terminal truncation mutants of gB and determined their cell surface expression, subcellular distribution, and fusion activity. Two mutants, including one that lacked the entire cytoplasmic tail domain, failed to function in the cell–cell fusion assay, despite wild-type levels of surface expression. This suggests that the OvHV-2 gB cytoplasmic tail is critical for fusion. A gB mutant truncated at amino acid 847 showed increased surface expression and fusion relative to the wild type. This suggests that the robust fusion activity of gB847 is the result of increased surface expression. gB847 may be used in place of wild-type gB in an improved, more robust OvHV-2 fusion assay.

## 1. Introduction

Ovine herpesvirus 2 (OvHV-2) is a gammaherpesvirus in the genus *Macavirus*, which is one of the most prevalent causative agents of malignant catarrhal fever (MCF). Sheep are a well-adapted carrier host and typically do not develop disease following infection. Infection in other, poorly adapted artiodactylid hosts, such as cattle and American bison, can result in sheep-associated (SA) MCF, which is a typically fatal disease characterized by severe lymphoproliferation and multisystemic vasculitis [1]. The virus exhibits tropism for CD8^+^ γδT cells, the primary cell type implicated in disease, and alveolar type II epithelial cells in sheep lungs in vivo [2,3,4,5]. There is currently no treatment or vaccine available for this disease. The viral envelope glycoproteins responsible for entry are potentially useful targets for interventions and thus are a significant focus of OvHV-2 research.

Herpesviral entry is mediated by the interactions of viral envelope glycoproteins with cellular receptors and the cellular membrane, either the plasma membrane or an endosomal membrane [6]. Glycoprotein B (gB), glycoprotein H (gH), and glycoprotein L (gL) are conserved among all herpesviruses and are necessary and sufficient for OvHV-2 fusion [7]. gB, a type I transmembrane protein and class III fusion protein, is considered the core herpesviral fusogen [8]. The primary function of gB is to interact with the host cell membrane and draw the membrane and the viral envelope together to facilitate fusion. gH and gL function as a heterodimer and must be co-expressed for their proper processing and trafficking [9]. The gH/gL heterodimer binds to a host cell receptor during fusion for many gammaherpesviruses [10,11,12,13] and is thought to have multiple functions in fusion, including interactions with gB [14,15,16,17,18].

**Figure 1:**
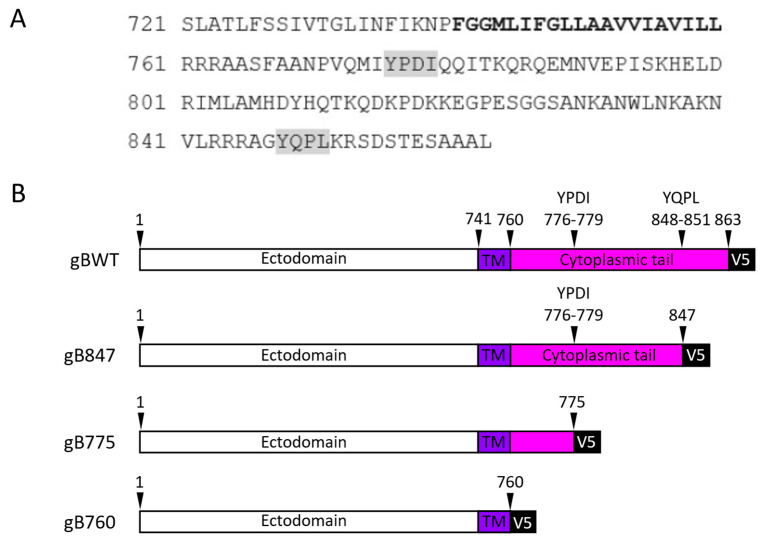
Schematics of OvHV-2 gB and truncation mutants. (**A**) Amino acid sequence of the C-terminus of OvHV-2 glycoprotein B. The predicted transmembrane domain (bold) spans amino acids 741–760. Two potential tyrosine-based endocytosis signaling motifs are shaded in grey. (**B**) Schematic representation of gB truncation mutants. Mutants are denoted by the amino acid length of the construct. TM, transmembrane domain (violet), cytoplasmic tail (pink).

Multiple functions have been attributed to the cytoplasmic tail of herpesvirus gB. Without the cytoplasmic tail, herpes simplex virus-1 (HSV-1) gB is misfolded and does not function in fusion [19]. Shorter truncations and point mutations of the cytoplasmic tail are also associated with syncytial strains of HSV-1 and -2 [20,21,22]. As such, this domain is thought to regulate fusion through a variety of mechanisms, including interactions with gH during fusion [23]. Of particular note, several motifs within this domain are conserved across herpesviruses, including putative tyrosine-based and dileucine endocytosis signaling motifs. Truncations or point mutations that affect these motifs modulate cell surface expression, which can correlate with the alteration of cell–cell fusion, although this relationship is not consistent. This effect has been described in the alphaherpesviruses HSV [20,24,25,26] and pseudorabies virus [27], the betaherpesvirus human cytomegalovirus [28], and, notably, the human gammaherpesviruses Epstein–Barr virus (EBV/HHV-4) [29,30] and Kaposi’s sarcoma-associated virus (KSHV/HHV-8) [31]. Specific functional domains have not been identified for OvHV-2 gB, including within the cytoplasmic tail. The OvHV-2 gB C-terminal tail includes two putative tyrosine-based endocytosis signaling motifs (Figure 1), which may affect surface expression and fusion.

OvHV-2 cannot be propagated in vitro, so an in-depth study of its infection cycle has proven difficult. Alternative strategies are imperative for understanding the fusion and entry mechanisms of OvHV-2. A virus-free cell–cell fusion assay is currently used to investigate the OvHV-2 membrane fusion reaction [7]. Such reporter assays of transfected cells are standard for dissecting the fusion activity of enveloped viruses [29,30,31,32,33,34,35,36,37,38]. Viral glycoproteins are expressed on the membrane of effector cells, which serves as a surrogate for the viral envelope. To accurately determine the fusion activity of a viral protein and the effect of mutations on fusion, the level of cell surface expression must be considered. Fusion activity is detectable in the OvHV-2 reporter assay, albeit weakly. A more robust assay is necessary to further understand the OvHV-2 fusion mechanism.

In this study, we provide evidence that truncating the cytoplasmic tail affects the surface expression of OvHV-2 gB in transfected cells. We also identify a truncated gB mutant (gB847) that triggers fusion at a significantly greater level than the wild type and exhibits a concomitant increase in cell surface expression. This form of gB with enhanced surface expression enables a more robust quantitative assay of OvHV-2 fusion.

## 2. Materials and Methods

### 2.1. Cells, Plasmids, and Antibodies

CHO-K1 cells (American Type Culture Collection (ATCC), Manassas, VA, USA) were propagated in Ham’s F12 Nutrient Mixture (Gibco/Life Technologies, Grand Island, NY, USA) supplemented with 10% fetal bovine serum (FBS) (Atlanta Biologicals, Atlanta, GA, USA) and 1× PSG. Immortalized sheep lung cells, A113 [7] were propagated in Minimum Essential Medium Alpha + GlutaMAX (Gibco/Life Technologies, Grand Island, NY, USA) supplemented with 10% FBS and 1× PSG.

The construction and sequences of plasmids encoding codon-optimized sequences for OvHV-2 glycoproteins B, H, and L [39] and empty vector pJP007 [7] were previously described. Plasmids encoding the firefly luciferase gene under T7 promoter control (pT7EMCluc) and the T7 RNA polymerase (pCAGT7) were also previously described [31,40].

OvHV-2 gB-specific monoclonal antibodies F1.2A and F2.15E were obtained from supernatants of selected hybridoma cell lines and quantified using an IgG (total) Mouse Uncoated ELISA Kit (Invitrogen). Hybridomas were previously produced via the intradermal inoculation of mice with the OvHV-2 gB expression plasmid (pOvHV-2-ORF8/V5) and selected based on OvHV-2 gB reactivity on ELISA, western blotting, and immunofluorescence assays [41].

### 2.2. Predicted Structural Model of OvHV-2 gB

Homology models of OvHV-2 gB in the pre-fusion and post-fusion conformation were built using the SWISS-MODEL server [42,43,44,45,46] with the OvHV-2 ORF8 sequence (protein ID ABB22229.1) as the input. This is the sequence of the virus obtained from the nasal secretions of sheep in Dubois, ID, USA [47]. The models were selected based on the highest sequence similarity of available homologs, total sequence coverage, and GMQE model quality score [45]. The ectodomain and transmembrane domains of the pre-fusion conformation model were modeled on the reported structure of HCMV gB (PDB: 7KDP) [48]. The cytoplasmic domain was modeled on the post-fusion structure of this domain in HSV-1 gB (PDB: 5V2S) [49]. For the post-fusion conformation, the ectodomain was modeled on the reported structure of KSHV gB (PDB: 9CU4) [50]. The transmembrane and cytoplasmic tail domains were modeled on the crystal structure of HSV-1 gB (PDB: 5V2S) [49].

### 2.3. Construction of OvHV-2 gB C-Terminus Cytoplasmic Tail Truncation Mutants

The OvHV-2 gB cytoplasmic tail domain (CTD) truncation mutants were created using the Q5 Site-Directed Mutagenesis Kit (New England Biolabs, Ipswich, MA, USA). The plasmids generated included pCL20 (gB847), pCL21 (gB775), and pCL22 (gB760) (Figure 1). Primers were designed based on the codon-optimized sequence of pOvHV-2-ORF8/V5 [39] as a template, using the NEBaseChanger primer design software v2.5.3 (New England Biolabs). The sense primer 5’-AAG GGT GGG CGC GCC GAC-3’ was used for all constructs. One antisense primer was designed for each construct: 5’-GCC TGC CCT TCT TCT CAG GAC GTT C-3’ (pCL20), 5’-GAT CAT CTG CAC GGG ATT AGC G-3’ (pCL21), and 5’-CAG CAG GAT CAC AGC GAT C-3’ (pCL22). A whole-plasmid sequencing analysis was performed to confirm the appropriate truncations in the CTD (Eurofins Genomic LLC, Louisville, KY, USA).

### 2.4. SDS-PAGE and Western Blot

**Figure 2:**
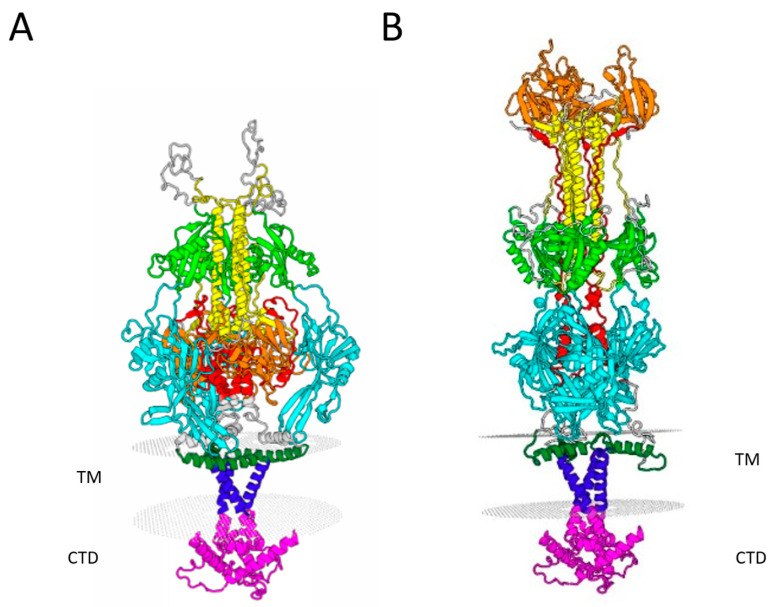
Structural models of OvHV-2 gB in (**A**) pre-fusion and (**B**) post-fusion conformations. Homology models were derived using SWISS-MODEL based on available published structures (see Materials and Methods). Domain I (cyan) is predicted to span residues 114–326; domain II (green) is predicted to span residues 103–113 and 327–418; domain III (yellow) is predicted to span residues 83–102 and 454–518; domain IV (orange) is predicted to span residues 518–625; domain V (red) is predicted to span residues 626–684; the membrane proximal region (dark green) is predicted to span residues 713–740; the transmembrane domain (violet) is predicted to span residues 741–760; and the cytoplasmic domain (pink) is predicted to span residues 761–863. TM, transmembrane. CTD, cytoplasmic domain.

**Figure 3:**
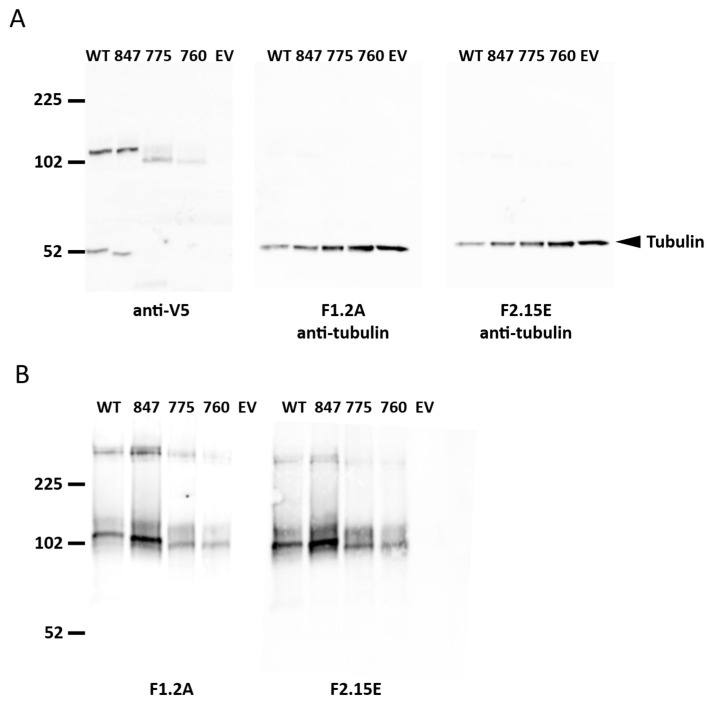
Expression and detection of OvHV-2 gB truncation mutants. Transfected CHO-K1 cell lysates were analyzed using (**A**) conventional (denaturing and reducing conditions) or (**B**) “native” SDS-PAGE, followed by western blot with anti-V5 or anti-OvHV-2 gB monoclonal antibodies F1.2A or F2.15E. Tubulin was used as a loading control in the middle and right panels of (**A**). Equal volumes of cell lysate were loaded for each lane. Molecular weight markers in kilodaltons are indicated to the left. EV, empty vector.

**Figure 4:**
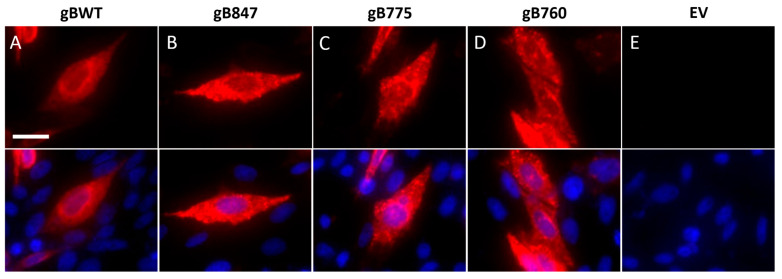
Microscopic detection of OvHV-2 gB truncation mutants in cells. Transfected CHO-K1 cells expressing (**A**) gBWT, (**B**) gB847, (**C**) gB775, or (**D**) gB760, or (**E**) those transfected with pJP007 empty vector (EV), were fixed with 3% paraformaldehyde and permeabilized with 0.2% Triton-X 100. OvHV-2 gB monoclonal antibody F1.2A was added and then detected with Alexa Fluor 647-conjugated goat anti-mouse antibody. Nuclei were counterstained with DAPI (overlay, bottom row). Magnification, 40×; scale bar, 20 µm.

CHO-K1 cells were transfected with plasmids encoding wild-type OvHV-2 gB (pORF8CUT-pcDNA3.2-V5, gBWT) or one of the generated truncation mutants: pCL20 (gB847), pCL21 (gB775), and pCL22 (gB760). Transfections were performed using the Lipofectamine 3000 kit (Invitrogen, Carlsbad, CA, USA). Cells were lysed 24 h post-transfection using 2% CHAPS in Hepes-buffered saline with EDTA-Free Halt Protease Inhibitor Cocktail (Thermofisher Scientific, Waltham, MA, USA). Samples for conventional (denaturing) conditions (Figure 2A) were prepared in sample buffer containing 2% SDS and 15 mg/mL DTT and boiled at 85 °C for 5 min. For “native” conditions, samples were prepared in sample buffer with 0.2% SDS and no DTT and were not boiled [51]. Transfected cell lysates in SDS sample buffer were separated on 8% (Figure 3A) or 4–20% (Figure 3B) tris-glycine gels (Invitrogen). Gels were transferred to a nitrocellulose membrane and then blocked with Pierce Protein-Free T20 TBS Blocking Buffer (ThermoFisher Scientific) for 30 min. Membranes were probed overnight with Anti-V5-HRP antibody (Invitrogen) at a 1:5000 dilution, OvHV-2 gB monoclonal antibodies F1.2A (0.47 mg/mL, 1:50 dilution) and F2.15E (0.51 mg/mL, 1:50 dilution), or anti-TUBA4A (TUBA1) antibody at 1: 10,000 (Sigma-Aldric, St. Louis, MO, USA). Goat anti-mouse IgG Alexa Fluor Plus 647 Secondary antibody (Invitrogen) was added for 30 min. Images were obtained using an Azure Biosystems Imager (Dublin, CA, USA).

### 2.5. Immunofluorescence Microscopy

CHO-K1 cells were seeded in a 24-well plate with a glass coverslip in each well 1 day prior to transfection. Cells were transfected with a plasmid-encoding gBWT, one of gB847, gB775, or gB760, or an empty vector plasmid pJP007 using the Attractene transfection reagent (Qiagen, Louisville, KY, USA) and incubated at 37 °C for 24 h. Following transfection, cells were washed with PBS and then fixed in 3% paraformaldehyde at 37 °C. To evaluate total cell gB expression, cells were permeabilized with 0.2% Triton-X 100 at room temperature. Coverslips were then blocked in 1% BSA PBS for 30 min, then incubated in a humid chamber with primary OvHV-2 gB-specific antibody F1.2A (0.39 mg/mL) at room temperature for 1 h. All coverslips were incubated in goat anti-mouse IgG Alexa Fluor Plus 647 Secondary antibody (Invitrogen) for 30 min and 5 ng/mL DAPI for 10 min. Coverslips were then mounted with Fluoromount G (ThermoFisher Scientific), and the slides were allowed to dry in the dark overnight. Images were obtained using the Leica DFC7000 T Fluorescence microscope at 10× and 40× magnification.

### 2.6. Flow Cytometry for Quantifying the Total and Surface Expressions of OvHV-2 gB

CHO-K1 cells were transfected as described above. Following transfection, cells were lifted using 0.1% Trypsin and then resuspended in 1× DPBS. A total of 5 × 10^5^ cells were aliquoted per well. Cells were fixed in 3% formaldehyde in PBS for 15 min at room temperature. To evaluate total protein expression, cells were permeabilized via incubation in 0.003% Triton-X 100 in 1% BSA for 10 min at room temperature. Primary antibodies OvHV-2 gB F1.2A (0.47 mg/mL) and F2.15E (0.051 mg/mL) were diluted at 1:10 and incubated on cells at room temperature for 30 min. Secondary antibody goat anti-mouse IgG Alexa Fluor 647 Plus (Invitrogen) was diluted at 1:200 and incubated on cells at room temperature for 15 min. Flow cytometry was performed using a CytoFLEX Flow Cytometer with CytExpert Software v2.5 (Beckman Coulter Inc., Brea, CA, USA). A total of 10,000 events were collected per condition. Data was analyzed using FCS Express v.6 Flow Research Edition (De Novo Software, Pasadena, CA, USA). Median Fluorescence Intensity (MFI), with the background (EV-transfected cells) subtracted, was used to quantify protein expression in permeabilized and non-permeabilized cells. One-way ANOVA with Dunnett’s test for multiple comparisons was used for statistical analysis.

### 2.7. Virus-Free Luciferase Reporter Assay for Cell–Cell Fusion

CHO-K1 (effector) cells were transfected with plasmids encoding T7 RNA polymerase (pCAGT7), OvHV-2 wild-type gH and gL, and one of gBWT, gB847, gB775, or gB760. Transfections were performed using the Attractene transfection reagent (Qiagen) and incubated for 24 h at 37 °C. A113 (target) cells were transfected with a plasmid encoding the firefly luciferase gene under the control of a T7 promoter (pT7EMCLuc). Transfections of target cells were performed using the Lipofectamine 3000 kit (Invitrogen) and incubated for 6 h at 37 °C. Following transfection, target cells were added to effector cells and co-cultured in MEM-Alpha medium for 18 h at 37 °C. Using the Luciferase Assay System (Promega, Madison, WI, USA), cells were frozen in 1× lysis reagent for 24 h; then, they were thawed, and the wells were scraped. Luciferase substrate was added to these cell lysates and assayed for light output (luciferase activity; fusion) using a BioTek Synergy HT microplate reader and Gen5 v3.08 software (Agilent, Santa Clara, CA, USA). One-way ANOVA with Šídák’s multiple comparisons test was used for statistical analysis.

## 3. Results

### 3.1. C-Terminally Truncated gB Mutants Are Expressed and Oligomerized in Transfected Cells

OvHV-2 gB is an 863 amino acid type I transmembrane glycoprotein. The predicted transmembrane domain is located at amino acids 741–760 [52]. The primary amino acid sequence of the OvHV-2 gB C-terminus includes two putative tyrosine-based endocytosis signaling motifs located at amino acids 776–779 and 848–851 (Figure 1). To evaluate the effects of the cytoplasmic tail of OvHV-2 gB on protein expression and fusion, we constructed three truncation mutants via the sequential deletion of the C-terminus. Two of the truncation sites were selected to eliminate one (gB847) or both (gB775) motifs. The third truncation mutant (gB760) eliminates the entirety of the predicted cytoplasmic tail. All constructs include a V5 epitope fused to the C-terminus of gB. The V5 epitope is widely used and is not anticipated to have any significant effects on gB structure, expression, or function due to its small size and neutral charge [53,54,55].

Herpesvirus gB is a class III fusion protein, together with vesicular stomatitis virus G and baculovirus gp64 [56]. The crystal structures of gB in the pre- and post-fusion conformation have been determined for several herpesviruses but are not yet available for OvHV-2 gB. To visualize the likely structure of this protein, we built homology models of OvHV-2 gB based on the available structures of related gB proteins using the SWISS-MODEL server [42,44]. Models were selected based on the highest sequence similarity of available homologs, the total sequence coverage, and the GMQE model quality score [45]. OvHV-2 gB exhibited the highest sequence identity (30.72%) with HCMV gB [48] among the homologs with pre-fusion structures available. Thus, OvHV-2 gB was modeled on this template (Figure 2A). No available pre-fusion conformation template included the cytoplasmic tail, so the post-fusion HSV-1 gB structure was used to model this domain, assuming that minimal conformational changes would occur within it. For the post-fusion structure, OvHV-2 gB exhibited the highest sequence identity (53.18%) with KSHV gB [50]. However, the available structure of KSHV gB includes only the ectodomain. The HSV-1 gB structure (25.64% sequence identity) [49] was used as a template to model the transmembrane domain and cytoplasmic tail. The two models were combined to produce a final model with maximum coverage of the OvHV-2 gB sequence (Figure 2B). The predicted pre-fusion conformation is a compact trimer consisting of five folded domains, including a long, central alpha helical domain (yellow, predicted residues 454–518). gB contains internal hydrophobic loops (two per monomer) that are pointed toward the membrane (predicted at residues 134–140 and 219–226). These function as fusion peptides for several gB orthologs. The post-fusion conformation is an extended, rod-like trimer with rearranged domains relative to the pre-fusion conformation.

Expression plasmids that encode the gB truncation mutants were transfected into CHO-K1 cells. gBWT and each of the gB mutants were expressed, as confirmed via western blot with anti-V5 antibody (Figure 3A, left panel). OvHV-2 gB was detected at approximately 110 kDa, which is the expected molecular weight of a gB monomer [39]. The C-terminal deletion mutants gB775 and gB760 migrated noticeably faster, which is consistent with the lower molecular weight of these constructs. There was an additional band at approximately 52 kDa for gBWT and gB847, as well as one of lower molecular weight for gB775. This band was absent for gB760. This likely represents a C-terminal fragment, which is predominantly composed of CTD. The two anti-OvHV-2 gB antibodies (F1.2A and F2.15E) tested did not detect gB in western blots, in which the lysate was treated under denaturing and reducing conditions (Figure 3A, middle and right panels). As such, we concluded that both antibodies target conformation-dependent, discontinuous epitopes on OvHV-2 gB.

The herpes fusion protein gB functions as a trimer, which is typically disrupted by detergent and heat [8,57,58,59]. To determine the effect of the truncation mutations on OvHV-2 gB oligomerization, transfected cell lysates were subjected to a more “native” SDS-PAGE analysis (no boiling, no reducing, and low SDS sample buffer). Following western blots with mouse monoclonal antibodies F1.2A or F2.15E (Figure 3B), ~110 kDa (monomer) and >225 kDa (trimer) gB species were detected for all wild-type and mutant constructs. This suggests that the conformation of the extracellular domain of the gB mutants is not significantly altered relative to the wild type at these epitopes. gB was not detected under standard denaturing PAGE conditions (Figure 3A, middle and right panels). Both antibodies reacted with the putative gB trimer, even in the absence of the entire cytoplasmic tail (gB760), suggesting that cytoplasmic tail domains are not required for OvHV-2 gB trimerization. In sum, gBWT, gB847, gB775, and gB760 are all expressed in transfected CHO-K1 cells and can be detected by OvHV-2 gB-specific antibodies.

### 3.2. Detection of Wild-Type OvHV-2 gB and Truncation Mutants in Transfected CHO-K1 Cells via Immunofluorescence Microscopy

We further evaluated the expression of gB and the truncation mutants within transfected cells using immunofluorescence microscopy (Figure 4). OvHV-2 gBWT and all three mutants were detected throughout the cell. gBWT, as well as gB847, gB775, and gB760, were distributed diffusely throughout the cell, with prominent perinuclear localization, as is typically described for gB of other herpesviruses [29,60] (Figure 4A). The faint nucleoplasmic signal is likely an artifact of epifluorescence imaging. Overall, these findings support the finding that the gB truncation mutants are expressed and widely distributed in transfected cells.

### 3.3. The OvHV-2 gB Cytoplasmic Tail Modulates Cell Surface Expression

**Figure 5:**
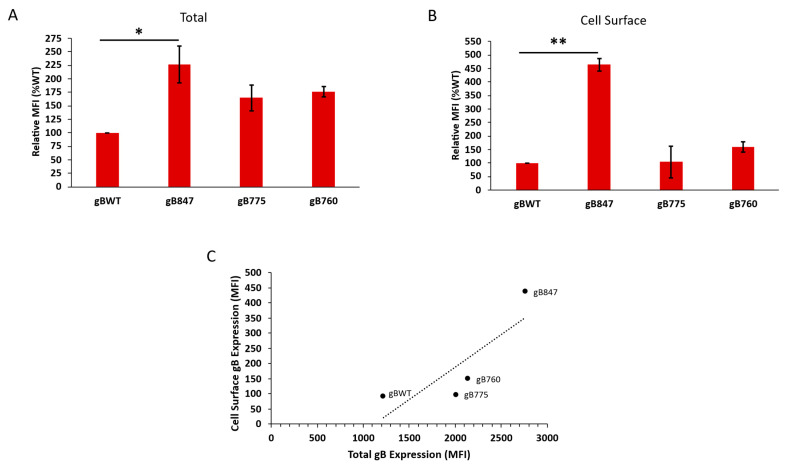
Total and surface expressions of OvHV-2 gBWT and the truncation mutants gB847, gB775, and gB760. Transfected CHO-K1 cells were fixed in 3% formaldehyde and either (**A**) permeabilized with 0.003% Triton-X 100 or (**B**) left untreated. Anti-gB antibodies F1.2A and F2.15E were added, followed by a fluorescent secondary antibody. The median fluorescence intensity (MFI) of gBWT was set to 100%. (**C**) Correlation of total and surface expressions of gBWT and each truncated mutant. Pearson correlation coefficient; r = 0.82, r^2^ = 0.68. Results are the mean of three independent experiments. *, *p* < 0.05; **, *p* < 0.01; One-way ANOVA with Dunnett’s test was used for multiple comparisons.

To quantify the surface expression of the gB mutants, we analyzed transfected cells using flow cytometry. Transfected CHO-K1 cells were fixed and either permeabilized to evaluate both intracellular and surface expressions or not permeabilized to evaluate surface expression only. The median fluorescence intensity (MFI) was calculated, and the background (MFI of empty vector-transfected cells) was subtracted. In the permeabilized cells (Figure 5A), the total expression of gB847, gB775, and gB760 was 2.3, 1.7, and 1.8 times greater than that of the wild type, respectively. This finding differs from the apparent relative gB expression levels on western blot (Figure 3B), but this disparity is attributed to key differences in the methodology. A combination of two anti-OvHV-2 gB primary antibodies was applied for flow cytometry rather than one for each western blot, thereby increasing sensitivity. However, cell permeabilization is imperfect and limits the penetration of the antibody into internal cellular components. The cell lysate used for western blot does not include the nuclear fraction; it also does not reflect the total gB content of the cell. As such, neither method perfectly detects total gB, and differences are expected. Strikingly, despite equivocal total protein expression compared to gBWT, gB847 was detected on the surface of the cell at 4–5 times the level of the wild-type protein (Figure 5B). The surface expressions of gB775 and gB760 were equivalent to that of gBWT. There was a positive correlation between the total and surface expressions of these proteins (Figure 5C); however, this correlation was imperfect (r = 0.82, r^2^ = 0.68), suggesting that there may be another mechanism that regulates surface expression.

### 3.4. The OvHV-2 gB Cytoplasmic Tail Includes Functional Domains, Which Limit or Are Required for Fusion

**Figure 6:**
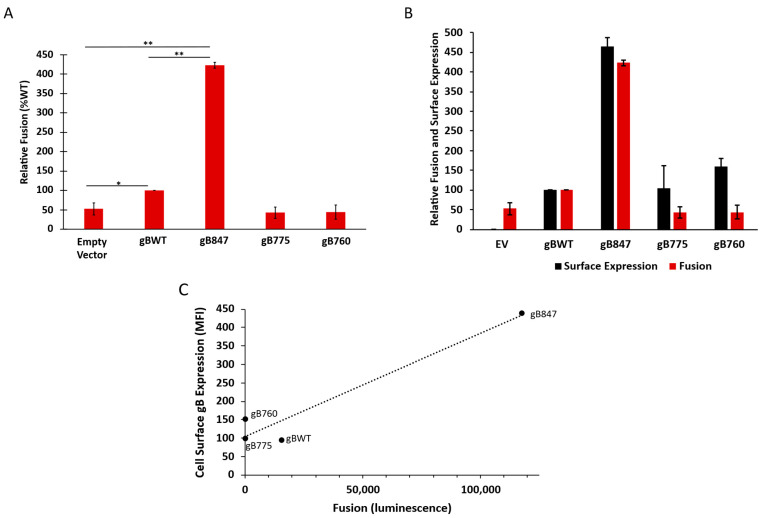
(**A**) Effect of OvHV-2 gB cytoplasmic tail length on fusion. Transfected CHO-K1 effector cells expressing gBWT, gB847, gB775, or gB760 with gH, gL, and T7 polymerase were co-cultured for 18 h with A113 target cells transfected with the luciferase plasmid. Luciferase-induced luminescence was detected as an indicator of fusion. Luminescence of gBWT fusion was set to 100%. Results are the means of three independent experiments. *, *p* < 0.05; **, *p* < 0.01; One-way ANOVA with Šídák’s multiple-comparison test. (**B**) Relative surface expression and fusion activity of OvHV-2 gB mutants. (**C**) Correlation of surface expression and fusion of gBWT and each truncated mutant. Pearson correlation coefficient: r = 0.97, r^2^ = 0.94.

We determined the effects of the gB cytoplasmic tail truncations on OvHV-2 fusion with a virus-free cell–cell fusion reporter assay (Figure 6A). Effector cells were transfected with equal quantities of plasmid DNA for gH, gL, T7 RNA polymerase, and gBWT or one of the three mutants. OvHV-2 wild-type gB, gH, and gL were required and sufficient for cell–cell fusion, as previously reported [7]. OvHV-2 fusion mediated by gBWT was specifically and reproducibly detected; however, the luminescence signal-to-empty vector background ratio was low and not ideal for analysis (approximately 2:1 at best). Fusion mediated by gB847 was approximately four times greater than gBWT, and notably 8–10 times greater than the background luminescence. In contrast, gB775 and gB760 did not mediate detectable fusion above the background, suggesting that they are fusion-dead.

We probed the relationship between cell surface expression and cell–cell fusion for each of the gB mutants. The transfection efficiency of all constructs was determined to be equivalent, as measured via flow cytometry (27–35%); thus, it was not considered to have a significant effect on the degree of fusion observed. When expressed as relative fusion and surface expressions (MFI), with the gBWT levels set to 100%, the increases in surface expression and fusion by gB847 were equivalent, suggesting that the robust fusion activity of gB847 is due to its increased surface expression (Figure 6B). In contrast, gB775 and gB760 exhibited wild type-like levels of surface expression but did not function in fusion, suggesting that these mutations greatly affected gB fusion function.

## 4. Discussion

Fusion is an essential step in herpesviral infections [61]. To study this mechanism in isolation, and because OvHV-2 cannot be propagated in vitro, the current best method for studying OvHV-2 fusion is a reporter assay for virus-free cell–cell fusion [7]. However, fusion in this assay for OvHV-2 is weak and insufficient to properly delineate details of the fusion mechanism. In this study, we demonstrate that the cytoplasmic tail of OvHV-2 gB is critical for cell surface expression and cell–cell fusion. We identify a truncated cytoplasmic tail mutant, gB847, that exhibits much more robust fusion. This form of OvHV-2 gB will be a valuable reagent for reliably measuring OvHV-2 fusion moving forward.

Glycoprotein B is the core herpesviral fusogen and is conserved across herpesviruses. OvHV-2 gB is homologous to gB in other herpesviruses, with a high sequence similarity and similar predicted structure in both the pre- and post-fusion conformations (Figure 2). As such, we predict that OvHV-2 gB will function similarly to the gB of other herpesviruses, including undergoing a similar conformational change. Alcelaphine herpesvirus 1 (AlHV-1), another virus in the *Macavirus* genus and the causative agent of wildebeest-associated MCF, is the most closely related herpesvirus for which gB has been studied in depth. AlHV-1 has been well studied, as it is a rare example of an MCF-associated virus that can be propagated in a cell culture [62]. gB from this virus has a similar molecular weight and is also cleaved into smaller fragments, including an approximately 50 kDa C-terminus fragment [63,64], which supports the homology of OvHV-2 with that of other herpesviruses.

The large N-terminal ectodomain of gB comprises five folded domains [8]. The primary role of gB is to undergo conformational changes within this ectodomain that drive the fusion of the viral envelope with a host cell membrane [65,66]. In addition to the ectodomain, the C-terminal cytoplasmic domain also influences a range of gB functions, including fusion [20,24,25,60,67,68,69,70,71,72,73,74,75,76,77,78,79,80,81,82,83,84]. In the human alphaherpesvirus herpes simplex virus 1 (HSV-1), the gB cytoplasmic tail has been shown to be required for normal gB folding and its interactions with the cytoplasmic tail of another core fusion protein, gH [14,23]. The interactions between the envelope glycoproteins are essential for herpesviral fusion and can affect the overall fusogenicity of a virus or a protein construct [16].

In addition to this finding, the cytoplasmic tail of gB is implicated in the intracellular trafficking and subcellular distribution of gB for multiple herpesviruses, including pseudorabies virus, human cytomegalovirus, human herpesvirus 6, and Epstein–Barr virus (EBV) [27,29,76,85]. The effects of the cytoplasmic tail on fusion and, particularly, on the robustness of cell–cell fusion assays have been well studied. For the human gammaherpesvirus EBV, a wide range of cytoplasmic tail mutants have been studied. The cytoplasmic tail of EBV gB modulates fusion activity [29], affects surface expression [30], and regulates the energy requirement for membrane fusion [86]. Similar effects on surface expression and fusion have been reported for the cytoplasmic tail of another human gammaherpesvirus, human herpesvirus 8 (HHV-8)/Kaposi’s sarcoma-associated herpesvirus (KSHV) gB [31]. Some of these effects have been attributed to endocytosis signaling motifs. Two putative endocytosis motifs were identified in the cytoplasmic tail of OvHV-2 gB (Figure 2A). These functions in EBV and HHV-8 have been used to improve the performance of cell–cell fusion assays. The frequency with which the cytoplasmic tail has been reported to affect fusion across alphaherpesviruses, betaherpesviruses, and gammaherpesviruses suggests that these are conserved functions.

This study represents the first functional analysis of OvHV-2 gB. We present evidence suggesting that the cytoplasmic tail of OvHV-2 gB is required for cell–cell fusion. Truncation mutants that lack most or all of the cytoplasmic tail (gB775 and gB760) do not function in fusion, despite wild type-like expression and cell surface exposure (Figure 6). The specific functional domains in the cytoplasmic tail and the role they play in fusion have yet to be determined, but these findings suggest that the cytoplasmic tail of OvHV-2 may share some of the essential fusion functions described for other herpesviruses.

The short truncation made in gB847 increases surface expression and fusion. Hyperfusogenic gB mutants exhibit a relative increase in fusion greater than any concurrent increases in surface expression [23,29,37,86], which is consistent with a change in gB function. Because the increased surface expression and fusion of gB847 relative to gBWT are equivalent (Figure 6B), the enhanced fusion activity of gB847 is likely the result of increased surface expression and is not suggestive of any significant gain of function. The function of this mutant gB allows for a more practical baseline for OvHV-2 cell–cell fusion. Thus, gB847 is ideal for use in a more robust OvHV-2 cell–cell fusion assay, thereby broadening the possibilities for further functional studies. This is particularly important given the absence of a viable culture system for OvHV-2.

## Data Availability

The raw data supporting the conclusions of this article will be made available by the authors on request.

## Abbreviations

The following abbreviations are used in this manuscript:

MCF	Malignant catarrhal fever
OvHV-2	Ovine herpesvirus 2
gB	Glycoprotein B
gH	Glycoprotein H
gL	Glycoprotein L
CTD	Cytoplasmic tail domain
EBV	Epstein–Barr virus
KSHV	Kaposi sarcoma-associated herpesvirus
HSV	Herpes simplex virus
